# Performance comparison of conductive textile electrodes in ECG monitoring

**DOI:** 10.3389/fbioe.2026.1772862

**Published:** 2026-02-27

**Authors:** Martin Lajdolf, Lukas Danys, Michal Prochazka, Daniel Debnar, Radek Martinek, Rene Jaros

**Affiliations:** Department of Cybernetics and Biomedical Engineering, Faculty of Electrical Engineering and Computer Science, VSB–Technical University of Ostrava, Ostrava, Czechia

**Keywords:** conductive textile materials, deep neural networks, electrocardiography, signal processing, textile electrodes, wavelet transform

## Abstract

The implementation of textile electrodes offers the possibility of improving comfort and reducing the risk of allergic reactions of the measured subject, while also opening the door to non-medical devices for everyday life. The main objective of the experiment is to test and compare five conductive fabrics manufactured by Shieldex in terms of the quality of the measured electrocardiography (ECG) signals. For this experiment, 3 electrodes of each conductive material were made and tested on a sample of 20 volunteers. The electrodes were attached by an elastic band at the locations of selected standardized ECG leads, and Ag/AgCl electrodes were used as reference. Correlation coefficient (R), signal-to-noice ratio (SNR) and percentage root-mean-square deviation (PRD) applied to representative cardiac periods were used to evaluate the quality of the acquired data. Differences in the results of the evaluation parameters between the different positions were noted, and when the distribution of positions was neglected, it was discovered that the best results were obtained with Shieldex Silitex material and, on the contrary, the worst results were recorded with Shieldex Technik-tex P130 + B material. It was also found that the results were not significant between the materials and after visual inspection it can be concluded that all materials are suitable for ECG measurements.

## 1 Introduction

Monitoring the electrical activity of the human heart is one of the most widely used diagnostic medical procedures. Systems mediating the acquisition of cardiac signals use different types of electrodes, including Ag/AgCl, balloon or clip electrodes, which may be associated with various disadvantages. One of the negative effects may be the risk of an allergic reaction (Kim et al., 2022), which is quite common with Ag/AgCl adhesive electrodes due to the use of adhesive and conductive gel. Another negative phenomenon related mainly to long-term cardiac monitoring is patient discomfort caused, for example, by the adhesive, which can cause discomfort even when removing the electrodes (Sundström et al., 2023). Furthermore, in the case of long-term monitoring, hydrogel electrodes are limited by the tendency of the gel to dry out, or, conversely, during stress measurements, they may begin to peel off due to increased sweat production on the subject being measured Yu et al. (2024). Dry conventional electrodes made of metals offer the great advantage of reusability, but often suffer from increased motion artifacts due to insufficient adhesion to the skin Wan et al. (2024). Textile electrodes have the potential to reduce the negative effects of conventional electrodes mentioned above (Ferri et al., 2022; Ravichandran et al., 2023). In this regard, textile electrodes appear to be an irreplaceable element for advanced epidermal systems Jeon et al. (2025). While the risk of allergy can also occur with textiles (Sanchez Armengol et al., 2022), it tends to be much smaller, nevertheless caution must be exercised when choosing a suitable conductive material. Many types of conductive textiles are now available, including textiles containing metallic filaments (Euler et al., 2022), textiles with printed conductive structures (Tu et al., 2023), or textiles containing conductive polymers (Zhou et al., 2021). The use of textile electrodes has potential not only in electrocardiography (ECG), but also in other medical fields such as electroencephalography (Rahman et al., 2023), electromyography (Ozturk et al., 2021), electrotherapy (Skrzetuska et al., 2021) or thermotherapy (Liu et al., 2024).

The use of textile electrodes not only for ECG purposes is very popular nowadays and quite a lot of studies are dealing with this issue. The topic of the study by Fink et al. (2021) is the design and development of sensor garments for ECG monitoring. The authors fabricated textile electrodes from silver-embroidered fabric and woven fabric containing silver, which they implemented in the chest panel of a T-shirt. Both types of electrodes were tested on a human subject, with visual inspection of the resulting signals (before and after pre-processing). It was found that the embroidered electrodes performed better than the woven electrodes, which, for example, exhibited greater instability in the DC component of the signal (Fink et al., 2021).

A study by Nigusse et al. (2020) discusses the design of a textile electrode for continuous ECG measurements. The authors chose a system of depositing a metal layer (Ag) on cotton and polyester by screen printing, with electrical resistivity of 1.64 $\Omega^{2}$ for cotton and 1.78 $\Omega^{2}$ for polyester. According to the authors, the textile electrodes captured clear R-peaks comparable to conventional electrodes in terms of amplitude characteristics, but a higher susceptibility to motion artfacts must be taken into account (Nigusse et al., 2020).

A study by Li et al. (2024) investigates various factors that affect the impedance at the electrode-skin interface on textile electrodes containing Poly(3,4-ethylenedioxythiophene):polystyrene sulfonate (PEDOT:PSS), during ECG scanning. In the experiment, 3 sizes (10, 20 and 30 mm circle plan), 3 shapes (triangle, square and circle) and the effect of contact area on impedance were compared and shown in graphs. In terms of size, the larger the electrode is, the lower the impedance it achieves; in the case of shapes, according to the graph, the impedance of the square electrode is higher than that of the other two shapes (the results change subtly with increasing frequency). In the case of partial contact, the impedance is significantly higher than that of the full contact electrode. The authors also compared the signal to noise ratio (SNR) of the different electrodes, and no differences were observed for the sizes and shapes. Significantly worse SNR results were observed in the case of partial electrode contact (Li et al., 2024).

In the case of textile electrodes, reusability is assumed, which raises the question of hygiene. A study by Doci et al. (2023) investigates the effect of washing and abrasion on textile electrodes. The authors used denim (cotton) as a base, followed by a PEDOT:PSS-based material and a conductive silicone-based material. In the experiment, electrodes with conductive embroidery, with PEDOT:PSS and with conductive silicone were tested. The results show that after 5 washing cycles, the specific resistance of the electrodes increased from 20 to 30 $\Omega^{2}$ to 25–40 $\Omega^{2}$ , in the case of abrasion testing, a value of 50 $\Omega^{2}$ (Doci et al., 2023) was reached.

A study by Alizadeh-Meghrazi et al. (2021) attempts to develop a standardized procedure for measuring impedance at the textile electrode-skin interface (agar model). Silver, carbon fiber, Polydimethylsiloxane (PDMS) based materials were used for the experiment, Ionic Liquids (IL) and PEDOT:PSS. The effect of size, contact pressure, ratio of the mentioned substances and long-term use was tested. The authors observed significant differences in impedance, but according to them, these differences are not of major importance on the quality of the signals obtained. In addition to impedance testing, ECG signals were also analyzed using parameters such as SNR. An interesting result is that the tested electrode, which achieved a very low impedance (comparable to Ag/AgCl), however, achieved a significantly lower SNR (Alizadeh-Meghrazi et al., 2021).

The SNR parameter was also used in a study by Hossain et al. (2022) that tested electrodes in the form of wristbands made by weaving, knitting and crocheting with yarns containing carbon nanotubes (CNT), cotton and thermoplastic polyurethane (TPU) in the first case and CNT, spandex and TPU in the second case. The authors used Ag/AgCl electrodes as reference electrodes for evaluation. The authors mention that the SNR of the signals from the textile electrodes was quite high, but the signals were sufficiently telling. Another parameter is the impedance of the electrodes and testing their resistance. In the case of impedance, the authors found that knitted electrodes had a lower impedance value than crocheted electrodes. The authors also found that the impedance decreases with increasing electrode size (7.1 cm^2^, 5.8 cm^2^, 3.2 cm^2^ used) and when compared to conventional electrodes, the impedance value is higher, but this reportedly does not directly affect their sensing performance. It has also been found that the impedance of textile electrodes decreases with increasing frequency. In terms of durability, the study found that after 10 wash cycles, the amplitude of the R and S waves decreased while the ECG features were still discernible (Hossain et al., 2022).

Studies focusing on the topics related to textile electrodes face several problems, including, for example, conducting the experiment on a limited number of subjects (often only the authors) or the lack of appropriate evaluation parameters (visual inspection, etc.). The goal of this experiment is to perform a comprehensive design, fabrication, and testing of textile electrodes on a larger sample of people using a commercial ECG monitor used in medical offices, followed by a statistical evaluation of the material that worked best. We had two main research questions. How does the type of conductive fabric affect the stability and quality of the measured ECG signal? Furthermore, are textile electrodes capable of preserving the morphology of the measured ECG signal (including testing individual parts of the cardiac cycle, i.e., P wave, QRS complex, and T wave) comparable to the ECG signal measured using conventional adhesive electrodes? This study is based on the hypothesis that the quality of ECG signals acquired using dry textile electrodes is primarily determined by the mechanical adaptability of the material, including properties such as thickness and elasticity.

The main contributions of the work are as follows:

- Testing of different textile materials for ECG monitoring.
- Tensile testing of used conductive textiles.
- Initial testing performed on synthetic data.
- Real dataset measured on 20 volunteers.
- Custom R-peak detector using a trained deep neural network (DNN) model.
- Quality assessment using correlation (R), signal-to-noise ratio (SNR) and percentage root-mean-square deviation (PRD).

The rest of the paper is structured as follows: Section Materials and Methods describes the overall methodology of the experiment and section Results contains a description and explanation of the results. Discussion and conclusion are included in last two sections.

## 2 Materials and Methods

This section deals with a detailed description of the measurement system used in this experiment, covering the whole process from data collection to data processing and preparation for analysis. It first discusses the design and calibration of the sensors that were used to capture relevant information from the experimental environment, and then describes the transfer of the measured values to the data acquisition system where they were collected and stored. The section also explains how the data was subsequently processed, including removing noise, correcting for outliers and providing validation, while also focusing on the methods of filtering, analysis and possible transformation of the signals. The measurement system was designed to ensure maximum accuracy and reliability of the results, and this section provides a detailed overview of all the key steps that led to correct and consistent data processing.

### 2.1 Textile electrodes

**FIGURE 1:**
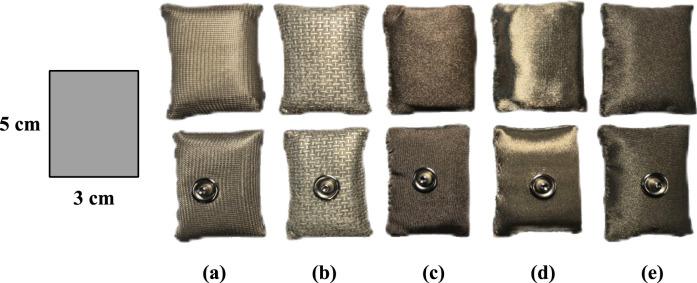
Photo of fabricated textile electrodes from front and back, **(a)** Balingen, **(b)** Bonn, **(c)** P130 + B, **(d)** P180 + B, **(e)** Silitex.

For this experiment, a total of five different materials were selected from the Shieldex manufacturer, which we used to make small pillows to maximize skin contact. This manufacturer is located in a close vicinity of our university and offers a suitable and wide selection of textile materials. Specifically, the conductive fabrics are Shieldex Balingen, Shieldex Bonn, Shieldex Technik-tex P130 + B, Shieldex Technik-tex P180 + B, Shieldex Silitex (referred to as Balingen, Bonn, P130 + B, P180 + B, Silitex). See the Table 1 for a description of the composition of these materials (median surface resistance measurement of the produced electrodes is also included) – additional information can be found in corresponding datasheets (Shieldex, 2024; Shieldex, 2024a; Shieldex, 2021; Shieldex, 2024b; Shieldex, 2022). The electrodes were manufactured by cutting each material into 6×5 cm pieces, which were carefully folded and then sewn into small pads. Press studs were then pressed into the resulting pockets to ensure a stable connection to the measuring chain. Memory foam was selected and custom-cut for pad filling to optimize skin contact and comfort. The inserts were secured with a concealed stitch using non-conductive thread. The resulting product are small pads of size 3×5 cm visible in Figure 1. In comparison, reference adhesive electrode had diameter of 3 cm with active area diameter of 1.7 cm.

**TABLE 1:** Overview of used conductive textiles.

Parameter	Balingen	Bonn	P130 + B	P180 + B	Silitex
Type of knit	Charmeuse	Non-woven	Stretch-tricot	Mirror-satin	Stretch-tricot
Metal	Ag	Ag	Ag	Ag	Ag
Material	(80% PA, 20% Ag) ± 10%	(83% PA, 17% Ag) ± 10%	(56% PA, 16% EL, 26.5% Ag, 1.5% coating) ± 10%	(68.7% PA, 4.4% EL, 25.4% Ag, 1.5% Coating) ± 5%	61.5% PA, 17.3% EL, 21.2% Ag, CSi coating (% not mentioned)
Electrical surface resistivity ( Ω /square)	0.6	0.5	2	2	5
Weight (g/m^2^)	62±10 %	56±15 %	132±18 %	205±15 %	380±10 %
Thickness (mm)	0.26±10 %	0.35±30 %	0.55±15 %	0.57±10 %	0.65±10 %
Temperature range ( ° C)	−30 to 90	−30 to 90	−30 to 90	−30 to 90	−30 to 90

Ag, Silver; CSi, Chlorinated silicones; EL, Elastomer; PA, Polyamide.

### 2.2 Dataset and experimental setup

**FIGURE 2:**
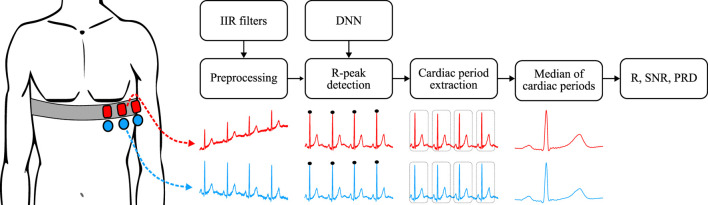
Schematic of the experimental steps, showing textile (red) and reference electrodes (blue) and their respective signals.

**FIGURE 3:**
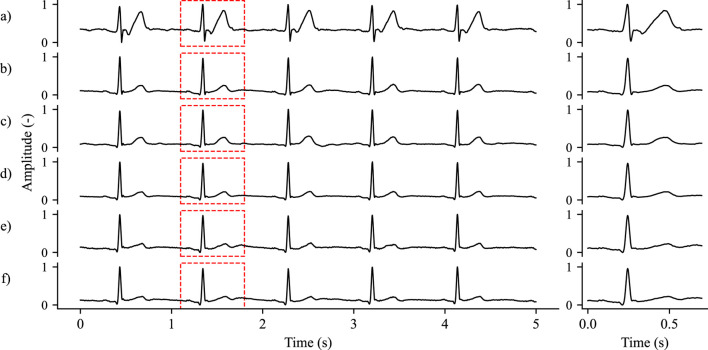
Graphical representation of normalized measured signals from Silitex material, subject 6: **(a)** Textile electrode V4; **(b)** Reference electrode V4; **(c)** Textile electrode V5; **(d)** Reference electrode V5; **(e)** Textile electrode V6; **(f)** Reference electrode V6.

The course of the experiment is visualized in the Figure 2. This experiment was divided into two parts. In the first part, the laboratory measurements on an ECG simulator (Ferronato FC12D) were performed by using one signal from this simulator, which was fed to two pieces of artificial skin. These parallel measurements ensured that both textile and reference electrodes (Ag/AgCl) can be used simultaneously. The generator was set to 60 beats per minute together with the generation of the usual artifacts such as zero line drift and noise. Real-time 12-lead ECG data was acquired using a portable commercial BTL Flexi 12 ECG device and transmitted via Wi-Fi to a dedicated desktop computer. The unfiltered signals were used for the experiment, with a sampling rate equal to 500 Hz. For preprocessing, two digital infinite impulse response (IIR) butterworth filters were used. First, a bandpass filter with a range of 0.5–100 Hz to filter out the high-frequency component, followed by a bandstop filter in the range of 49–51 Hz to remove network interference (50 Hz), with the orders of both filters taking the value of 2. The second part of the experiment involved obtaining real data on live subjects. Approval to perform the experiment on human subjects was granted by the Ethics Committee of the Faculty of Electrical Engineering and Computer Science of VSB-TUO under the following reference number VSB/23/109804. All methods were performed in accordance with the relevant guidelines and regulations. Informed consent was obtained from all subjects. A total of 20 subjects of variable ages (18–72 years) and somatotype participated in the experiment, with adequate representation of sexes. The sample size was determined based on state-of-the-art practice with the aim of exceeding the standard commonly used in the studies analyzed. By testing several electrode samples on each subject, this range ensures high statistical power. A total of 5 measurements (one for each textile type) of 6 min duration were performed on each volunteer. Standardized lead positions V4, V5 and V6 shown in the Figure 2 were used for every scenario. Real data was acquired using the same BTL equipment as for synthetic data. A Philips abdominal belt was used to ensure optimal contact of the tested electrodes. The reference electrodes were placed in the vertical axis in close proximity to the textile electrodes. The visualization of the signals obtained from the textile and reference electrodes is shown in the Figure 3. Signals measured for this experiment on volunteers are available on the IEEE DataPort (Jaros, 2025a).

### 2.3 Evaluation parameters

**FIGURE 4:**
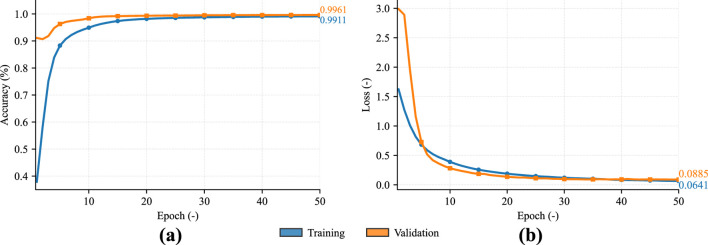
Graphical representation of the training process of the DNN model for R-peak detection. **(a)** Accuracy; **(b)** Loss.

Correct detection of R-peaks was essential for the evaluation. Detection is based on 2D processing of the ECG signal, which is converted to scalograms using continuous wavelet transform (CWT) and then segmented by a DNN into QRS complexes and signal residues. The trained model generates continuous probability series for classes Q, R, and S, with the final identification of the R-peak being performed by finding local maxima and minima in the individual probability series. The position of the R-peak itself is then defined using thresholding. Finally, verification is performed based on correlation, where a signal excerpt is made for each detected peak and compared to the median of these excerpts. The DNN model utilized for this experiment was trained and validated on a total of two datasets, one was measured at VSB-TUO as part of research into textile electrodes: to test the effect of the placement of textile electrodes on signal quality Jaros (2025b). Second dataset is divided into 2 subdatasets Matonia et al. (2020) called Pregnancy and Labour contains abdominal ECG (aECG) signals, which are essentially a combination of maternal and fetal ECG data. Selected aECG signals were used to train this model, from which maternal ECG (mECG) signals were obtained using extraction algorithms. The training progress, including accuracy and loss can be seen in the Figure 4. Based on these detections, individual cardiac periods were extracted in each signal as a (R-peak - 250 ms to R-peak +450 ms) (Sarafan et al., 2020). For each signal, a median cardiac period (MCP) was calculated and then normalized.

To evaluate signal quality, the agreement between reference and processed signals is often assessed using statistical metrics. The correlation coefficient, signal to noise ratio (SNR) and the percentage root-mean-square deviation (PRD) are among the most important parameters, each providing a unique perspective on signal quality. To ensure a comprehensive evaluation of the quality of measured signals, it is advisable to apply multiple evaluation parameters that provide different perspectives on the issue. While the correlation coefficient measures the similarity of signal shapes and the relationship between them, differences in amplitudes are not reflected in the result. While SNR evaluates the quality of the signal above the noise, PRD normalizes the error relative to the signal energy.

The Pearson´s correlation coefficient (R) can be used to evaluate the strength and direction of the linear relationship between two vectors (signals). It takes values from −1 to 1, with −1 indicating a perfect negative correlation, 1 indicating a perfect positive correlation and 0 indicating that there is no linear relationship between the vectors. The method does not take into account the magnitudes of the vectors under study, so theoretically no normalization is necessary for R in the case of this experiment. Pearson’s correlation coefficient was performed according to the following Equation 1:

$$R_{xy}=\frac{\sum_{i=1}^{n}\left(x_{i}-\bar{x}\right)\cdot \left(y_{i}-\bar{y}\right)}{\sqrt{\sum_{i=1}^{n}{\left(x_{i}-\bar{x}\right)}^{2}}\cdot \sqrt{\sum_{i=1}^{n}{\left(y_{i}-\bar{y}\right)}^{2}}},$$ (1)

where $x_{i}$ represents the part of the signal under test, $\bar{x}$ represents the average of the part of the signal under test, $y_{i}$ represents the part of the reference signal, $\bar{y}$ represents the average of that part of the reference signal, and n represents the number of samples of which the parts of the signals are composed (Tjøstheim et al., 2022).

The signal-to-noise ratio parameter is a quantity that defines the quality of a recording. SNR logarithmically compares the power of the useful signal (reference signal) and the power of unwanted components, or noise (textile signal–reference signal). The resulting value is given in decibels (dB) and indicates how much higher the level of the useful signal component is than the unwanted components containing noise and other artifacts. The higher the SNR parameter value, the better the result achieved by the textile electrode. In the context of this experiment, the SNR application was performed according to the following Equation 2:

$$SNR_{dB}=10\log_{10}\left(\frac{\sum_{i=1}^{n}x_{i}^{2}}{\sum_{i=1}^{n}{\left(x_{i}-y_{i}\right)}^{2}}\right),$$ (2)

where $x_{i}$ is the i-th sample of the Ag/AgCl signal, $y_{i}$ is the i-th sample of the textile signal, and n represents the total number of signal samples.

The percentage root-mean-square deviation parameter can be used to quantify the degree of signal distortion. This evaluation parameter compares the shape of the obtained test signal to the gold standard, i.e., in the context of this experiment, to the signal obtained from the reference electrode. The result of the parameter is given in %, indicating the extent to which the signal character deviates from the ideal signal. In this case, the rule is that a lower value indicates a better result. The PRD parameter can be calculated using the following Equation 3:

$$PRD=100\cdot \sqrt{\frac{\sum_{i=1}^{n}{\left(y_{i}-x_{i}\right)}^{2}}{\sum_{i=1}^{n}x_{i}^{2}}},$$ (3)

where, as in previous cases $x_{i}$ is the i-th sample of the Ag/AgCl signal, $y_{i}$ is the i-th sample of the textile signal, and n represents the total number of signal samples.

## 3 Results

This section deals with the interpretation of the results of this experiment, which was divided into two parts. In the first part, the textile electrodes were tested on synthetic data, and the results were used to assess whether their functionality is sufficient and whether it is appropriate to proceed with the experiment on real subjects. This process enables the comparison of textile electrode behavior on both real-world and synthetic data. The second part deals with testing electrodes on real data. For this purpose, median segments containing the P wave, QRS complex, and T wave were created in parallel with individual MCPs to reveal whether textile electrodes affect the shape and size of individual segments.

### 3.1 Experimental results on synthetic data

The evaluation parameters R, SNR, and PRD were used to evaluate the pre-processed synthetic data. An analysis of the R-peak amplitudes was also performed, based on which the parameter of the amplitude difference between the MCP of the examined material and the reference electrode was defined. Looking at Table 2 containing the results of this analysis, it can be seen that the evaluation parameters R, SNR, and PRD are consistent for each material. In the case of R, it was necessary to interpret the results to a total of seven decimal places in order to record the differences between the individual materials. The P130 + B fabric achieved the best result (R: 0.9999935, SNR: 48.8295 dB, and PRD: 0.3820%). A slightly worse result was achieved by material P180 + B (R: 0.9999929, SNR: 48.4641 dB, and PRD: 0.3866%), which also recorded the lowest difference in R-peak amplitude compared to the reference value (0.0026 mV). The second lowest R-peak amplitude difference value is for Silitex (0.0040 mV), but it is important to note that the results for this parameter are very similar for all parameters (with the exception of Balingen fabric). The analysis also included individual segments containing the T wave, QRS complex, and P wave. Looking at the table, it is clear that the P wave achieved the worst results in all materials. Conversely, the QRS complex achieved the highest consistency.

**TABLE 2:** Results for synthetic data experiment.

Material	Segment	R (−)	SNR (dB)	PRD (%)	R-peak amplitude (mV)
Ag/AgCl	-	-	-	-	1.0631
Balingen	MCP	0.9999709	41.6359	1.0233	1.0700
	P	0.9997820	33.5819	2.7634	
	QRS	0.9999865	43.8198	0.6282	
	T	0.9999427	39.4057	1.5808	
Bonn	MCP	0.9999786	43.4533	0.8697	1.0674
	P	0.9998731	35.9488	2.3428	
	QRS	0.9999844	44.8658	0.5594	
	T	0.9999716	42.2432	1.2979	
P130 + B	MCP	0.9999935	48.8295	0.3820	1.0589
	P	0.9999396	39.1622	1.0178	
	QRS	0.9999974	52.8971	0.2203	
	T	0.9999865	45.5279	0.6014	
P180 + B	MCP	0.9999929	48.4641	0.3866	1.0605
	P	0.9999467	39.7041	0.9489	
	QRS	0.9999967	51.7599	0.2528	
	T	0.9999841	44.9622	0.5864	
Silitex	MCP	0.9999879	46.0975	0.5563	1.0671
	P	0.9999331	38.7265	1.3708	
	QRS	0.9999913	47.4152	0.4340	
	T	0.9999812	44.2080	0.7218	

**FIGURE 5:**
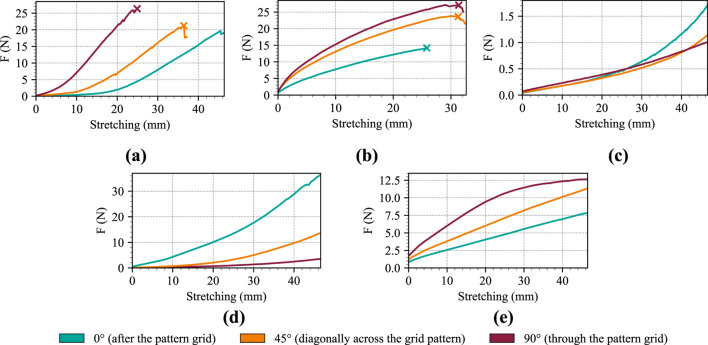
Illustration of tensile test curves for individual materials for various cuts: **(a)** Balingen, **(b)** Bonn, **(c)** P130 + B, **(d)** P180 + B, **(e)** Silitex.

All materials were subjected to tensile tests to determine their mechanical resistance. For the purposes of the test, material samples measuring 6.5×1 cm were prepared, with the test area measuring 5.5×1 cm (0.5 cm on both sides was inside the jaws of the testing device). The samples were cut in three different patterns: cut along the fabric structure (hereinafter 0° ); cut diagonally across the fabric structure (hereinafter 45° ); cut perpendicular to the fabric structure (hereinafter 90° ). This test focused on measuring both the elongation of the material and the electrical resistance, which was constant at 0 Ω for all materials until breakage occurred. The test results are shown in the Table 3, which contains information about the total elongation of the material, the force required to achieve this, and whether the material broke. The tensile test curves are also shown in the Figure 5, which depicts the elongation curves of the materials together with the point of breakage, indicated by the symbol × .

**TABLE 3:** Results of tensile tests on conductive materials.

Material	Cut	Force (N)	Length (mm)	Outcome
Balingen	0°	18.15	46.50	Heavily stretched
	45°	21.19	36.41	Ruptured
	90°	26.35	24.93	Ruptured
Bonn	0°	14.19	25.84	Ruptured
	45°	23.28	31.28	Ruptured
	90°	27.09	31.37	Ruptured
P130 + B	0°	1.71	46.50	Severely stretched
	45°	1.15	46.50	Severely stretched
	90°	1.01	28.85	Slightly stretched
P180 + B	0°	36.33	46.50	Severely stretched
	45°	13.64	46.50	Heavily stretched
	90°	3.54	46.50	Slightly stretched
Silitex	0°	7.85	46.50	Fully recovered
	45°	11.34	46.50	Fully recovered
	90°	12.66	46.50	Fully recovered

Figure 5 clearly shows that different cuts of materials have a noticeable effect on elasticity and resulting tensile strength in most cases. In the case of Balingen material, the least elastic cut was 90° , with samples breaking on average after less than 26 mm. Slightly higher elasticity was observed for the 45° cut, with samples beginning to break after approximately 36 mm. The 0° cut was the only one that did not break and achieved the maximum elongation with the lowest force applied. The Bonn material achieved a longer overall elongation, with all cuts eventually breaking. The 0° sample achieved the worst result, requiring significantly less force to break than the other two types of cuts, whose results were similar. The P130 + B material achieved the best elasticity result of all materials. All cuts achieved maximum elongation with a force of only up to 1.7 N (0°) , which is a fraction compared to other materials. In addition, the 90° cut achieved the least damage and returned to almost its original state after the test was completed. In the case of P180 + B fabric, there are significant differences in the force required to achieve maximum stretch, with a difference of around 33 N between the 90° and 0° cuts. It can therefore be concluded that the effect of the cut is most pronounced in this material. The last material, Silitex, showed high elasticity, with a force ranging from approximately 8–13 N required to stretch the samples to their maximum position. It should also be noted that all samples returned to their original state after the test, indicating that Silitex is a durable material in this test.

**FIGURE 6:**
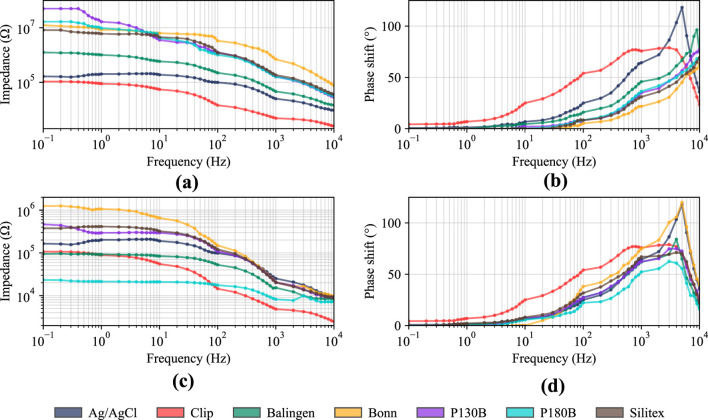
Impedance testing of conductive textiles: **(a)** Impedance without conductive gel; **(b)** Phase shift without conductive gel; **(c)** Impedance with conductive gel; **(d)** Phase shift with conductive gel.

The experiment also included testing the impedance (Z) at the electrode-skin interface. Material samples with a contact area of 1×1 cm were attached to the inner part of the wrist at a distance of 5 cm from the edges of the contact areas. The measurement was performed using the voltage divider method with bipolar connection. Frequency impedance dependencies were ensured by logarithmic scaling with steps ranging from 0.1 Hz to 1 kHz. Figure 6a shows the results of the dry electrode-skin impedance experiment, where it is apparent that the Balingen material is closest to the properties of the clip (dry reference) electrode. The overall trend of gradually decreasing impedance with increasing frequency can also be seen in Figure 6c, which describes measurements simulating a wet environment (sweat), where conductive gel was added to the samples using a pipette. In the case of a wet environment, a steeper decline in Z from a frequency of 100 Hz and overall lower Z values compared to measurements without gel can also be seen. In addition, the two conductive fabrics (P130 + B and Balingen) showed lower Z at all tested frequencies than the wet reference electrode (Ag/AgCl). Figure 6b describes the dependence of the phase shift (φ) on frequency in dry conditions, while Figure 6d describes the same quantities in wet conditions. At first glance, it is apparent that in both cases, the phase shift began to rise sharply at approximately 2 Hz. In the dry environment, φ reached its maximum at 10 kHz. In contrast, in the wet environment, φ reached its maximum between 4 and 5 kHz, with a very sharp drop recorded at a frequency of 10 kHz.

### 3.2 Real data experiment results

**FIGURE 7:**
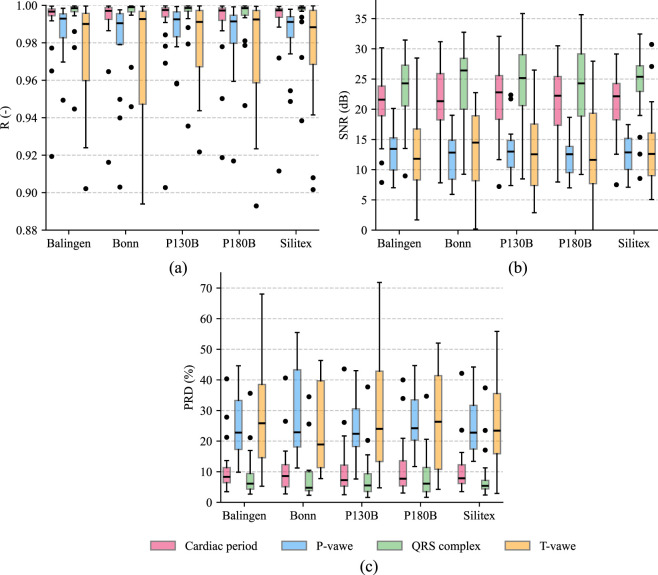
Box plot results of real data for the V6 lead: **(a)** Correlation, **(b)** SNR, **(c)** PRD.

The experimental results for the real data are interpreted in three tables and box plots. In the Table 4, the median values of the correlations for each material for each electrode position can be found. The same applies to Table 5 containing values for the SNR parameter and Table 6 containing the resulting values for the PRD parameter. The last column in these tables contains the median of the individual values when the positions are neglected, i.e., the median of the values for all positions. In the case of both figures, only the values for electrode position V6 were selected. The Figure 7a contains a box plot of the correlation while the Figure 7b contains a box plot of the SNR and the Figure 7c contains a box plot of PRD values. In all graphs, each material is represented by 20 values (1 value corresponds to one volunteer) for the standardized position V6. Comparison of the three tables reveals substantial differences in individual positions. The superior R, SNR and PRD values for lead V6 likely result from its proximity to the left ventricle and closer alignment with the cardiac axis.

**TABLE 4:** Resulting median R for real data.

Position	Segment	Balingen	Bonn	P130B	P180B	Silitex
V4	MCP	0.9556	0.9225	0.9495	0.9597	0.9413
	P	0.9644	0.9633	0.9632	0.9770	0.9728
	QRS	0.9632	0.9611	0.9596	0.9725	0.9661
	T	0.9893	0.9810	0.9915	0.9916	0.9925
V5	MCP	0.9911	0.9947	0.9936	0.9938	0.9930
	P	0.9889	0.9899	0.9853	0.9888	0.9901
	QRS	0.9955	0.9971	0.9966	0.9962	0.9964
	T	0.9943	0.9929	0.9949	0.9875	0.9930
V6	MCP	0.9967	0.9969	0.9976	0.9973	0.9974
	P	0.9929	0.9904	0.9925	0.9914	0.9911
	QRS	0.9985	0.9990	0.9986	0.9985	0.9987
	T	0.9900	0.9927	0.9911	0.9924	0.9883
V4-V6	MCP	0.9884	0.9901	0.9866	0.9876	0.9919
	P	0.9898	0.9847	0.9842	0.9884	0.9884
	QRS	0.9936	0.9935	0.9938	0.9943	0.9944
	T	0.9906	0.9915	0.9921	0.9918	0.9926

**TABLE 5:** Resulting median SNR for real data (values given in dB).

Position	Segment	Balingen	Bonn	P130B	P180B	Silitex
V4	MCP	9.2620	7.9400	8.2517	9.0899	7.6345
	P	7.2976	7.1625	7.5054	8.6143	6.4636
	QRS	10.8643	9.8982	10.1298	11.0823	9.9814
	T	6.5134	4.9859	5.1348	7.6221	6.4595
V5	MCP	17.2538	19.6751	18.8327	18.9706	17.7021
	P	9.5411	9.6749	10.7009	9.0373	10.0869
	QRS	20.2181	22.1767	21.6247	21.0136	21.4224
	T	10.4426	11.9194	11.3467	10.9392	12.3160
V6	MCP	21.5863	21.3375	22.8015	22.2385	22.1463
	P	13.4393	12.8252	12.9951	12.5535	12.8640
	QRS	24.2897	26.4178	25.1637	24.2867	25.3763
	T	11.8054	14.4796	12.5494	11.6170	12.6083
V4-V6	MCP	15.5298	16.8185	15.1181	15.3779	17.2518
	P	10.0482	9.3766	10.1927	10.6273	9.8665
	QRS	18.8806	18.8643	18.8257	18.7448	19.0893
	T	9.3844	10.3124	10.0208	10.0304	11.0253

**TABLE 6:** Resulting median PRD for real data (values given in %).

Position	Segment	Balingen	Bonn	P130B	P180B	Silitex
V4	MCP	34.4279	40.1403	38.6765	35.1823	41.5355
	P	43.4833	43.8417	42.2820	37.2160	47.5279
	QRS	28.6429	32.0126	31.2619	27.9880	31.9185
	T	47.2658	56.5269	56.0538	41.6853	47.9678
V5	MCP	13.7325	10.4033	11.5230	11.2620	13.0294
	P	33.3616	33.0922	29.2197	35.7354	31.3228
	QRS	9.7837	7.7856	8.3020	8.9027	8.4951
	T	30.0671	25.3600	27.1715	28.4606	24.2752
V6	MCP	8.3364	8.5970	7.2514	7.7292	7.8536
	P	22.7859	22.8930	22.4034	24.1999	22.7660
	QRS	6.1054	4.7783	5.5208	6.1059	5.3863
	T	25.8307	18.9011	24.0119	26.3318	23.4248
V4-V6	MCP	16.8185	14.4281	17.7997	17.1059	13.7334
	P	32.2611	34.3908	30.9741	29.5065	32.4093
	QRS	11.3788	11.4402	11.5065	11.5600	11.1072
	T	33.9720	30.5263	31.5922	31.5233	28.1262

This section describes the results of individual MCP segments and is structured into sections devoted to individual evaluation parameters (R, SNR, PRD). The results of the Pearson correlation coefficient can be seen in Table 4, which shows at a glance that the overall trend in signal quality gradually improves from position V4 to V6. In the case of lead V4, the highest R values were recorded for the T wave in all materials. In the case of the QRS complex and P wave, the results are similar with slight differences. The lowest result for the entire cardiac period was achieved with the Bonn material (R = 0.9225). For lead V5, the QRS complex achieves the best R values for all materials, while the worst values were recorded for the P wave. The worst result for MCP was achieved with the Balingen material. Overall, the highest R values were recorded at position V6, where the T wave was evaluated as the segment with the lowest R values in the MCP itself, while the QRS complex, similarly to V5, achieved the highest R values. When disregarding the distinction between positions (V4-V6), it can be seen that the overall correlation trend of the individual MCP segments is similar to position V5. The lowest values were achieved for the P wave, while the QRS complex achieved the highest results. In the case of the evaluation of the entire MCP, Silitex achieved the highest correlation (R = 0.9919). For a better visual representation of the R results, Figure 7a was created, containing the R values for position V6 (outliers specified in Table 7).

**TABLE 7:** Outliers overview for box plots (the limits of the axes have been adjusted in the figures for clarity, some outliers are beyond these limits).

Metrics	Segment	Balingen	Bonn	P130B	P180B	Silitex
R (Figure 7a)	MCP	3	2	4	3	2
	P	1	0	2	2	2
	QRS	3	2	3	3	4
	T	1	1	1	1	3
SNR (Figure 7b)	MCP	2	0	1	0	1
	P	0	0	1	0	0
	QRS	1	0	0	0	3
	T	0	0	0	0	2
PRD (Figure 7c)	MCP	3	2	2	2	2
	P	2	2	2	2	2
	QRS	2	2	2	1	3
	T	2	0	2	1	0
Total number of samples for each box				20		

The results of the SNR evaluation parameter can be found in Table 5 and, for a better visual representation, also in Figure 7b, which shows a box plot of the SNR results for position V6. The table shows that the overall difference between the individual positions is quite significant: the SNR for QRS for position V4 is 10 dB on average and for position V6 it is 24 dB on average. In the case of position V4, the best results were achieved between the individual MCP segments in the QRS complex, while the lowest SNR values were recorded for the T wave. The highest values of the entire MCP were achieved by the Balingen material. In position V5, the highest results among the segments were also achieved in the QRS complex, while the lowest values were recorded for P waves (very similar to T waves), with the highest SNR values of the entire MCP recorded for the Bonn material. Position V6 shows a similar trend for individual MCP segments as V4: the highest SNR values were recorded for QRS complexes and, conversely, the lowest for T waves. The highest SNR for the entire MCP was recorded for the P130 + B material. When combining the results of the electrode positions on the body, the lowest results were recorded for the P and T wave segments, while the highest SNR was achieved by the QRS complexes. Overall, the highest result for the entire MCP was achieved by the Silitex material with an SNR value of 17.2518 dB.

The PRD parameter and its results are recorded in Table 6. The V4 position showed the highest values of this parameter overall. In the case of individual MCP segments, the lowest values were achieved in QRS complexes, while the highest values were recorded in T waves. The lowest value of the entire MCP was recorded in the Balingen material. For position V5, the lowest value of the MCP segment analysis was also recorded in QRS complexes, while the highest values were recorded in P waves. The material whose MCP achieved the lowest PRD for the V5 position is Bonn. The V6 position shows the lowest PRD values of the entire MCP, while in the case of the analysis of individual segments, the lowest values were achieved by QRS complexes, while T waves achieved the highest PRD. The material with the lowest PRD of the entire MCP is P130 + B. For the summary position V4-V6, the lowest PRD values were recorded for QRS complexes, while the highest values were recorded for P and T waves. In the analysis using the PRD parameter, the lowest overall result for this parameter was recorded for Silitex material (PRD = 13.7334%). Results for V6 position is also showed in Figure 7c.

## 4 Discussion

The main objective of this experiment was to comprehensively verify the suitability of five selected conductive textiles for ECG measurement. It also sought to determine whether Silitex, with its properties such as high elasticity, durability, and low skin irritation, would achieve high results in a series of tests that the materials underwent in this experiment and would be a suitable candidate for further testing. In previous section, the results for individual textile electrode positions were interpreted even if these positions were neglected, i.e., only the median values of individual materials were taken. Overall, Silitex performed best all evaluation parameters (R: 0.9919, SNR:17.2518, PRD: 13.7334). These results indicate high similarity between signals measured with the presented textile and conventional electrodes even though the V4 position was included in the evaluation, it achieved significantly lower results than the V5 and V6 positions. Looking at the Figure 7a, it can be seen that outliers occur only in the direction of deterioration of the given evaluation parameter. This phenomenon is caused by the high quality of the results. Thus, if, for example, the median correlation value for all materials is higher than 0.99, it is not possible to find an outlier approaching 1 with this data variability. For even better interpretation of the results, it would be appropriate to use statistical tests. During the experiment, the non-parametric Mann-Whitney test was applied due to the failure to achieve normal distribution of the data by Shapiro–Wilk test. However, the test results did not confirm statistically significant differences between the individual groups at the significance level of α=0.05 . Additionally, a comparison and definition of this experiment in relation to other studies is provided in Table 8. The studies compared are listed and described in the introductory section.

**TABLE 8:** Overview of studies on textile electrodes.

Authors	Research area	Evaluation parameters	Dataset	Conclusion
Fink et al. (2021)	Textile electrode T-shirt testing	Visual	1 healthy individual	The embroidered electrode shows better signals
Nigusse et al. (2020)	Testing of silver-printed electrodes on cotton and polyester textiles	Surface resistance, SNR	1 healthy individual	Signals from textile electrodes are comparable to concentration methods
Li et al. (2024)	Investigation of the effect of size, shape and contact of PEDOT:PSS electrodes on impedance	Skin-electrode impedance, SNR	1 healthy individual	Impedance lower for larger electrodes, higher for poor contact, as well as SNR, which is not affected by size and shape
Doci et al. (2023)	Resistance to washing and abrasion	Specific resistance	1 healthy individual	Increase in resistance after 50 wash cycles, also after abrasion
Alizadeh-Meghrazi et al. (2021)	Different material ratios, resistance, impedance	Impedance, SNR	Agar skin model	Impedance has no effect, fabrics have potential
Hossain et al. (2022)	Weaving, embroidery and knitting testing	SNR, impedance, durability	Not specified	High SNR, impedance decreases with increasing area, after 10 duty cycles amplitude changes
Proposed	Testing of 5 conductive textiles	R, SNR, PRD, surface resistance, electrode-skin impedance	ECG generator and 20 individuals of variable somatotype, age and sex	Electrode resistance does not affect the signals, best results: Silitex, no significant differences were observed

For the purpose of this experiment, the positions of the standardized leads V4, V5 and V6 were chosen in order to test the electrodes as close as possible to the heart of the subject. By looking at the Tables 4–6, it can be seen that the median values show clear differences between the locations, especially for the V4 location, which differs significantly from the other two locations. Visual inspection of the signals revealed that several subjects showed noticeable differences between the textile and reference signals, such as a decrease in the amplitude of the R-wave, or conversely, an increase in the amplitude of the S-wave in the case of the textile electrodes. This phenomenon may be caused by several reasons. The first factor is the anatomy of the human’s body itself. There is much a larger layer of fat or muscle around V4 position, in comparison to position V6, which may affect the presence or absence of various interferences of the ECG signal. Another important factor is the size of the electrodes themselves. Theoretically, the electrodes sense biological signals as a matrix system. By this we mean that an electrode can be interpreted as a set of quantum “microelectrodes” that sense signals from a quantum of points on the human skin. The resulting signal is therefore the sum of the signals of the individual points of electrodes. This theory raises the possibility of incorrect textile electrode sizing, particularly at V4 position. While the electrode may have been positioned correctly, it might also have captured signals from other locations, masking or drowning the intended V4 lead signal. As already mentioned, a visual inspection of each signal was performed and the mentioned phenomenon of changes in amplitude magnitude occurred mainly in subjects on the borderline of malnutrition, suggesting that the size of the textile electrode may have had a negative effect. In this regard, the next phase of the experiment, building on the results of this pilot study, will focus on evaluating the size of the textile electrodes, their placement on the body, the method of connection to the measurement system, and their robustness against motion and other artifacts. With reference to Figure 3, a significantly greater T-wave amplitude and a marginally increased S-wave amplitude are observed for the textile electrode at position V4 when compared to the signal obtained using the reference electrode. This phenomenon was observed across all tested materials in subject 6 and was also noted in several other subjects whose somatotype can be classified as borderline malnutrition. This fact, together with the previously mentioned factors, may play a key role in the production of this phenomenon. It is also necessary to consider the possibility of measurement error, which may have been caused by a slight deviation from the intended axis of measurement. This phenomenon can theoretically be eliminated by measuring the reference and textile electrodes at the same location, which is, however, impossible when measuring with both electrodes at the same time. It would certainly be worthwhile to test this phenomenon in a further experiments to evaluate the effect of the size and placement of the textile electrodes for ECG sensing.

The presented combination of algorithms and their settings is based on our earlier research, which can be traced back to articles and studies from reputable research teams. Additional information regarding the evaluation and signal processing is in our earlier article focusing on vectorcardiography transformations (Jaros et al., 2019).

Part of these studies (Introduction section) mentioned that textile electrodes were more susceptible to noise and motion artifacts. During the presented experiments there was no significant increase in noise for textile electrode signals compared to Ag/AgCl signals. Figure 2 shows the real signals for position V5. It is apparent that the RAW signals from both types of electrodes are noisy at similar intensities, noting the presence of a more noticeable zero-line drift in the signal from the textile electrode. After preprocessing, the signals are virtually identical (see Figure 3 for preprocessed textile and reference signals at all positions). During data acquisition, it was discovered that the tested electrodes are noticeably more susceptible to the formation of motion artifacts in comparison to Ag/AgCl electrodes. Speech and deep breathing caused artifacts across all electrodes, but with greater intensity in the textile signals. While tightening the chest band effectively reduced these artifacts, it negatively impacted comfort, which is the main advantage of textile electrodes. Future studies should evaluate the electrodes during activities such as walking or weight-bearing exercise, and explore optimal elastic band pressure to balance both artifact reduction and patient comfort.

When testing the impedance of conductive textiles at the electrode-skin interface in a dry environment, it is immediately apparent that Balingen is the only conductive textile to achieve the lowest impedance values, standing out from the other materials in the graph. In Table 1, which interprets the data sheets of the textiles used, Balingen material is defined as a charmeuse fabric that exhibits extreme softness. This softness allows the samples to achieve high mechanical conformity, creating a number of contact points with high pressure on the skin, which increases adaptability to skin irregularities. Looking at the graph showing the relationship between frequency and phase shift, it is also apparent that the same fabric achieves the highest phase shift of all fabrics. In wet testing, the impedance is significantly lower, which may be due to the filling of air cavities between individual fabric fibers with a conductive gel, which also helps the fabric to compensate for skin irregularities. Figure 6c shows a very interesting result: the P180B material achieves significantly lower results than the reference wet electrode. The result can be attributed to the fact that, thanks to its properties, the material achieved a larger effective contact area than the reference. When comparing the figures showing the relationship between frequency and phase shift, the difference between the dry and wet scenarios is again visible, with the maximum phase shift for most electrodes being achieved at 4–5 kHz in wet testing (compared to around 9–10 kHz in dry testing). The results of impedance and phase shifts indicate that in the case of conductive textile materials tested in this experiment, the presence of sweat does not necessarily pose a problem causing errors in conductivity and artifacts. On the contrary, human sweat (saline solution) can help increase the sensory properties of textile electrodes, which can assist in the selection of suitable material, for example, for a sports vital signs monitor, which will certainly be exposed to increased perspiration over a long period of time.

Analysis of Tables 4–6 reveals that all materials performed very well at the V6 lead position, achieving high similarity to the reference signals. When choosing the right material, the sensing capability is of course the most important parameter that should guide the system designer. There are also other very important aspects on the basis of which such a designer might decide to choose this particular material. How is the material handled? What is the durability? Is it comfortable to wear? Can the material be sanitized or washed in any way? Working with the Balingen material was quite challenging. It is a charmeause fabric (Shieldex, 2024) that has a very soft and flexible texture, which made it difficult to sew into electrodes. After testing the electrodes on 20 subjects, it is also possible to notice some wear in the seams of the electrodes, which is due to the flexibility mentioned above. In the case of this material, the use of a non-conductive fabric as a lining could be considered to increase the overall strength. On the other hand, it should be mentioned that the material is extremely comfortable and the manufacturer also declares washability. Another material is Bonn, which is classified as a non-woven fabric (Shieldex, 2024a). The fabric is not flexible and there were no problems in the manufacture of the electrodes. This material did not experience the problems encountered on Balingen, but degradation of the electrode surface was nevertheless observed. The material also did not produce discomfort for the subjects, but it is certainly worth noting that the manufacturer does not recommend washing it at all. According to the manufacturer, P130 + B is a stretch-tricot material (Shieldex, 2021), the texture of which is somewhat similar to that of Balingen, so that the manufacturing of the electrodes was subtly more complex. Lining could be also used to improve the overall strength of the electrodes. The measured subjects reported that it was not an extremely comfortable material, but at the same time they were not uncomfortable. Interestingly, there were virtually no signs of wear and tear after the experiment and the washability is also a significant advantage. According to the manufacturer, the P180 + B material is classified as a mirror-satin fabric (Shieldex, 2024b). Given the minor manufacturing issues with this fine material, a lining could offer a possible solution. P180 + B is probably the most comfortable material used in this experiment. Some of the subjects on whom measurements were taken even reported that they would not mind wearing a shirt made entirely of this material. A slight disadvantage is the slight wear (puckering) of the material in the seam areas, which is not as noticeable as in the Balingen material. The manufacturer also declares that this material can be washed. The last material tested is Silitex, classified as a stretch tricot knit fabric (Shieldex, 2022). This type of material is characterized by high elasticity, a fine structure that is relatively comfortable on human skin, and good mechanical resistance. What makes Silitex unique is that one side of the knit fabric is coated with a layer of conductive silicone, which significantly enhances the overall strength of the electrode and improves the stability of the molded contact used to connect it to the measuring chain during design and production. In the case of this experiment, it is undoubtedly the fabric that was the best to work with. There were no problems around the seams when sewing the electrodes. There was also virtually no wear and tear on the electrodes after the experiment. The only slight disadvantage may be that, for example, compared to P180 + B material, it is not as comfortable a textile, but on the other hand, none of the subjects complained of discomfort. Another advantage of Silitex is its washability. Wear and tear and the possibility of sanitation or any kind of hygienic operation with these materials were also topics of some of the studies mentioned. An important observation of this study is the fact that no adverse skin reactions were observed with any of the tested textile electrodes, while mild skin irritation was reported in some subjects at the sites where the reference electrodes were attached. In the future, it would also be useful to test these particular materials, particularly in the context of sanitation and their wear and tear or resistance to human sweat.

Although advances in materials engineering have introduced very promising alternatives to conductive textiles, such as carbon-based materials, PEDOT:PSS coatings, or other modern systems Lee et al. (2025), our study focused exclusively on silver-plated textiles. While new materials such as liquid metals offer excellent conformability and lower skin-electrode interface impedance due to their softness Ma et al. (2024), silver-containing textiles currently represent a robust commercially available solution for mass production of smart clothing due to established manufacturing processes, cost-effectiveness, and washability. The main objective of this study was to reveal whether these commercially produced fabrics are suitable candidates for smart clothing systems and whether Silitex, with its properties, will dominate in selected samples. The aim was not to evaluate experimental prototypes. However, future studies should certainly take these materials into account and perform in-depth comparative tests.

A very important part of this experiment is the actual creation of representative cardiac periods, which requires accurate definition of the R-peak positions. As mentioned in section Materials and Methods, a custom detector was used to detect R-peaks in the signals. The proposed detector is based on a DNN processing 2D CWT scalograms, which shows significantly higher success rates compared to, for example, the Pan-Tompkins algorithm. The main drawback of the Pan-Tompkins algorithm was its susceptibility to errors in cases where the amplitude of the S wave exceeded the amplitude of the R wave (low R wave and deep S wave), or where T or P waves were misclassified as R peaks due to amplitude thresholding. In contrast, the proposed detector radically reduced this negative phenomenon by using signal segmentation and subsequent logical evaluation and validation. Thanks to the strict requirement that the R-peak be located between the predicted Q and S waves, the system uses the morphological context of the entire QRS complex. While Pan-Tompkins detectors rely on fixed mathematical heuristics that can fail with highly variable signals, neural networks offer a solution capable of adapting to complex anomalies, provided they are trained on a sufficiently diverse and comprehensive dataset. It is also important to note that although the detector constructed for use in this experiment is significantly more robust than the conventional algorithm mentioned above, it is far from flawless. The trained model is still in the early stages of development, which will continue for a long time. The model was trained on approximately 100 h of diverse ECG signals, including pathological phenomena. In the future, we plan to significantly expand the training dataset, primarily with signals contaminated by various types of noise or other artifacts. This procedure will make it possible to further improve the overall detection of R-peaks and prevent false detections in problematic signals. Another future goal is to adapt the model to abdominal ECG signals, which contain both the mother’s ECG and the fetal signal. This topic involves very complex processing of abdominal signals from which fetal signals are extracted. With a suitably trained model, it is possible to skip the complex extraction of fetal signals and detect both maternal and fetal R-peaks at the same time. Figure 4 shows the training process of the DNN model. The curves show that the validation accuracy (val. ACC) is higher than the training accuracy (train ACC). At first glance, this phenomenon may look like an error, but it is a logical phenomenon. Dropout layers were applied during the model design, which essentially randomly turn off some neurons, increasing the model’s generalization ability by preventing unwanted co-adaptation of neurons. During the validation process, these dropout layers are turned off, often resulting in a better ACC result than during training. In the case of the loss function, the opposite effect can be seen: the validation loss function (val. loss) is higher than the training loss function. This is caused by the strong weighting of minority classes (Q-wave, R-peak, S-wave), which penalizes the loss function even with very low uncertainty in the model prediction.

The potential of textile electrodes lies in several fields of electrocardiography (and not only ECG). This experiment focuses on testing electrodes contained in an elastic chest belt, and although only positions from 3 standardized leads were tested here, the idea of the experiment is a system containing textile electrodes (ideally a chest belt) that can be used for long-term clinical ECG imaging. Such a system should provide several advantages, including ease of installation on the patient, comfort of wear and repeatability of use. A similar system is addressed, for example, in the study by Teferra et al. (2022). Another attractive application is the implementation of textile electrodes in clothing, which is addressed, for example, in the study by Fink et al. (2021). Conversely, these systems may have benefits in non-ambulatory applications, which may include commercial tools for biosignal measurement. The concept of an autonomous sports T-shirt capable of capturing ECG, respiratory activity, and other biosignals during various physical activities and transmitting them wirelessly to a smartphone is an attractive approach to this problem.

## 5 Conclusion

This study focused on the sensory evaluation of five materials from the manufacturer Shieldex, which were used to make textile electrodes for ECG measurements. To obtain the dataset, measurements were performed on twenty subjects of variable gender, age and somatotype. The electrode placement was fixed to the positions of the standardized ECG leads (V4, V5, V6), and the textile electrodes were attached using an elastic band, while reference electrodes (Ag/AgCl) were glued directly under the textile electrodes. Evaluation of signal quality was performed based on correlation coefficient, signal-to-noise ratio and percentage root-mean-square difference. Based on the obtained results, it was found that all materials showed very high similarity to the reference data. It was also found that the quality of the signals increases significantly when approaching the V6 position. When the positions of electrodes were neglected, it was found that the P130 + B material achieved the worst results while the Silitex material achieved the best results. Based on these preliminary findings, it can be concluded that materials tested in this study can be used for the fabrication of textile electrodes, but other aspects affecting data acquisition, such as electrode size and placement, must also be considered an explored.

## Data Availability

The datasets presented in this study can be found in online repositories. The names of the repository/repositories and accession number(s) can be found below: https://ieee-dataport.org/documents/electrodes-conductive-textile-materials.
